# Zelda overcomes the high intrinsic nucleosome barrier at enhancers during *Drosophila* zygotic genome activation

**DOI:** 10.1101/gr.192542.115

**Published:** 2015-11

**Authors:** Yujia Sun, Chung-Yi Nien, Kai Chen, Hsiao-Yun Liu, Jeff Johnston, Julia Zeitlinger, Christine Rushlow

**Affiliations:** 1 Department of Biology, New York University, New York, New York 10003-6688, USA;; 2 Stowers Institute for Medical Research, Kansas City, Missouri 64110, USA;; 3 Department of Pathology and Laboratory Medicine, Kansas University Medical Center, Kansas City, Kansas 66160, USA

## Abstract

The *Drosophila* genome activator Vielfaltig (Vfl), also known as Zelda (Zld), is thought to prime enhancers for activation by patterning transcription factors (TFs). Such priming is accompanied by increased chromatin accessibility, but the mechanisms by which this occurs are poorly understood. Here, we analyze the effect of Zld on genome-wide nucleosome occupancy and binding of the patterning TF Dorsal (Dl). Our results show that early enhancers are characterized by an intrinsically high nucleosome barrier. Zld tackles this nucleosome barrier through local depletion of nucleosomes with the effect being dependent on the number and position of Zld motifs. Without Zld, Dl binding decreases at enhancers and redistributes to open regions devoid of enhancer activity. We propose that Zld primes enhancers by lowering the high nucleosome barrier just enough to assist TFs in accessing their binding motifs and promoting spatially controlled enhancer activation if the right patterning TFs are present. We envision that genome activators in general will utilize this mechanism to activate the zygotic genome in a robust and precise manner.

After fertilization, the genome of a zygote is initially quiescent, but transcription soon begins in a precise temporal manner, first with a small subset of early genes followed by hundreds to thousands of genes (for review, see Tadros and Lipshitz 2009; Lee et al. 2014). This process of zygotic genome activation (ZGA) was thought to be under the control of many TFs with different roles in embryogenesis, but with the discovery of the “genome activators” in *Drosophila* (Vfl/Zld) (Liang et al. 2008) and zebrafish (Nanog, Pou5f3, and SoxB1 family factors) (Lee et al. 2013; Leichsenring et al. 2013), it was realized that a single factor, or a small group of factors, could play a global role in genome activation. These factors bind to specific sequence motifs prior to ZGA (Harrison et al. 2011; Leichsenring et al. 2013); and in *Drosophila*, there is a striking correlation between Zld motifs and the magnitude and timing of zygotic gene expression during ZGA (Liang et al. 2008; Harrison et al. 2011; Nien et al. 2011; Satija and Bradley 2012). Thus, these factors are thought to prime the genome for subsequent transcriptional activity. Here, we focus on *Drosophila* ZGA and the mechanism by which Zld primes enhancers for genome activation.

Zld is detected in nuclei before 1 h post-fertilization, as early as nuclear cycle 2 (nc2) (Nien et al. 2011), thus long before patterning TFs such as Dl (nc9) (Rushlow et al. 1989; Kanodia et al. 2009; Liberman et al. 2009) and Bicoid (Bcd, nc6) (Little et al. 2011). When the patterning TFs become active after 1 h, they bind in a pattern that correlates with their cognate motifs only when Zld motifs are present nearby (Satija and Bradley 2012). Furthermore, deleting Zld motifs in select Twist (Twi), Dl and Bcd enhancers in transgenic reporter assays resulted in decreased TF binding (Yáñez-Cuna et al. 2012; Foo et al. 2014; Xu et al. 2014), and in the case of the *sog* enhancer, decreased DNase I hypersensitivity (Foo et al. 2014). These observations suggest that Zld facilitates TF binding by promoting chromatin accessibility. For these reasons, Zld is described as a “pioneer” factor (Harrison et al. 2011; Foo et al. 2014; Li et al. 2014), a special class of TFs that access the genome first and promote chromatin accessibility for other TFs (for review, see Zaret and Carroll 2011; Iwafuchi-Doi and Zaret 2014). However, how Zld regulates chromatin accessibility is not known.

Here, we utilized chromatin immunoprecipitation and micrococcal nuclease digestion followed by sequencing (ChIP-seq and MNase-seq, respectively) to examine the global role of Zld in shaping the enhancer chromatin landscape to potentiate TF binding.

## Results

### Zld promotes Dl binding to developmental enhancers

Our previous transgenic analysis showed that Zld potentiates Dl binding to the enhancers of its targets *brk* and *sog* (Foo et al. 2014). To examine the role of Zld in mediating Dl binding genome-wide, we performed ChIP-seq with anti-Dl antibodies on chromatin from 2–3 h wild-type and *zld*^−^ embryos (see Methods for validation of *zld*^−^). Since *zld*^−^ embryos show extreme disorganization at the end of nc14 due to lack of cellularization and nuclear fallout (Liang et al. 2008), we stained fixed wild-type and *zld*^−^ embryos with DAPI and removed disorganized, as well as out-of-stage embryos from all experiments. This ensures that wild-type and *zld*^−^ populations are homogenous, and the phenotype we observe is due to loss of Zld rather than secondary effects.

**Figure 1.**
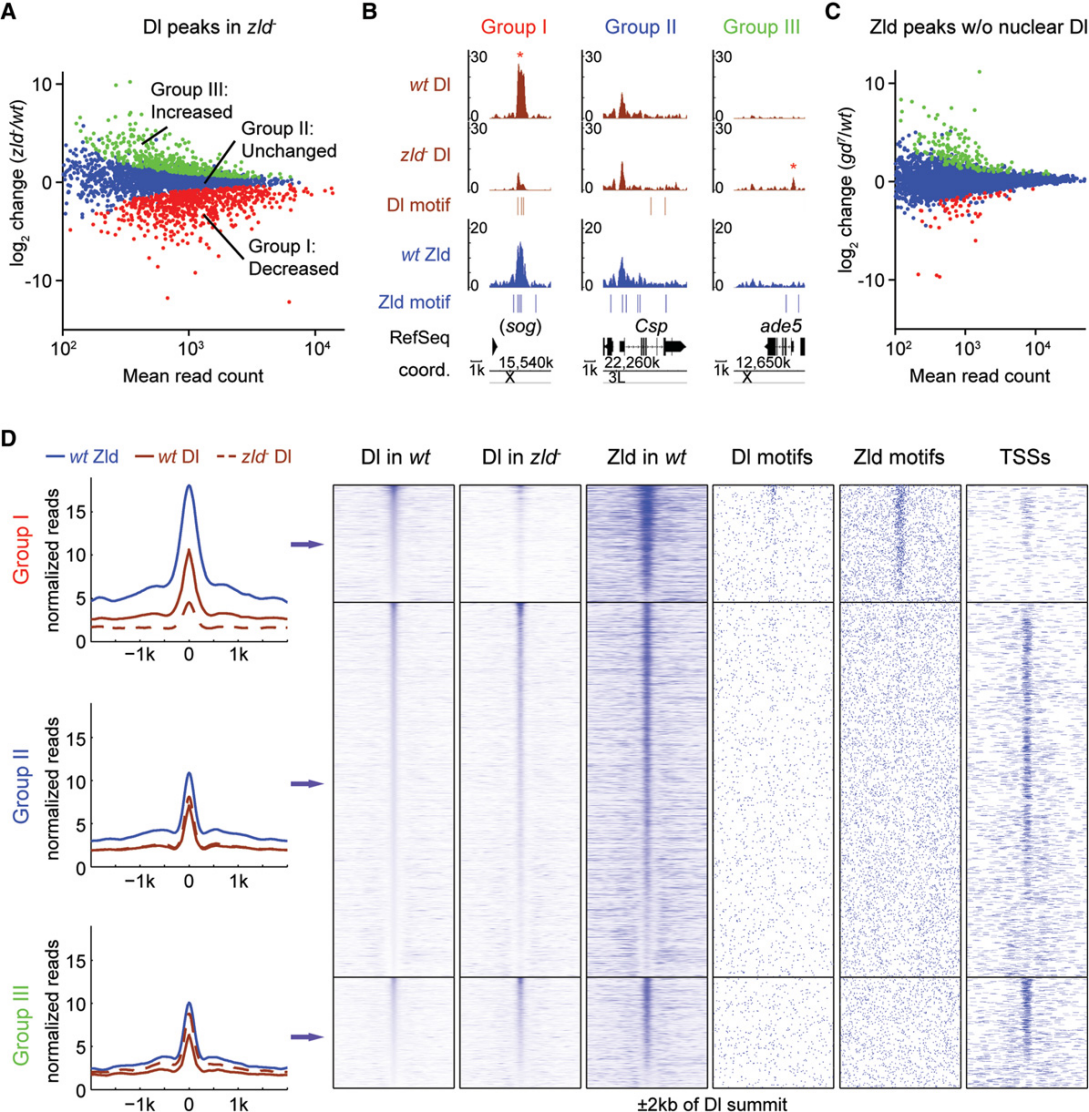
In the absence of Zld, Dl is lost at developmental enhancers and redistributes to accessible regions such as promoters. (*A*) MA plot of differential Dl binding in *zld*^−^ versus wild-type (*wt*) embryos. The *x*-axis represents the mean of normalized Dl reads per peak; the *y*-axis represents the log_2_ fold-change of normalized reads per peak between the genotypes. Significantly decreased peaks (Group I, red), not significantly changed peaks (Group II, blue), and significantly increased peaks (Group III, green) were identified by DESeq with FDR < 0.1. (*B*) Integrated genome browser (IGB) views showing examples of Dl peaks (brown tracks) within the three Dl-peak groups (*y*-axis represents normalized read counts), together with nearby Zld binding (blue track), as well as Dl and Zld motifs. For the *sog* locus, the shadow enhancer that lies ∼20 kb upstream of the TSS is shown (Hong et al. 2008). Asterisks denote peaks significantly changed in *zld*^−^. (*C*) MA plot as shown in *A* of differential Zld binding in *gd*^*7*^ (in which no Dl activation occurs) versus *wt* embryos. The number of significantly changed peaks is much smaller. (*D*) Metaprofiles and heatmaps of normalized Dl and Zld ChIP reads for each Dl-peak group, along with nearby Dl and Zld motifs, and annotated TSSs. All regions are centered on the Dl summit (in *wt*) and extend to each side by 2 kb. Note that *wt* Dl binding within Groups II and III is on average much lower than within Group I. Note also that only Group I is highly enriched for Dl and Zld motifs and has significantly less TSSs than the other two groups, consistent with the hypothesis that they are enriched for enhancer regions.

Using MACS (Zhang et al. 2008), we identified a total of 3499 Dl peaks across both genotypes (see Methods). We next utilized the DESeq package (Anders and Huber 2010) to look for differential Dl binding between wild-type and *zld*^−^ embryos. At an FDR < 0.1, 679 (19.4%) Dl peaks were found significantly decreased in *zld*^−^ (referred to herein as Group I) (Fig. 1A), which is in accordance with the predicted role of Zld in facilitating Dl binding to the genome; for example, Dl binding at the *sog* enhancer is significantly reduced in *zld*^−^ (Fig. 1B). This decreased Dl binding is not due to a change in Dl protein levels, because the Dl gradient is both quantitatively and qualitatively comparable between wild-type and *zld*^−^ (Foo et al. 2014). Of the remaining Dl peaks, 2176 (62.2%) exhibited insignificant differential binding between genotypes (Group II), whereas 644 (18.4%) Dl peaks significantly increased in *zld*^−^ (Group III), suggesting that Dl binding redistributes in the absence of Zld (Fig. 1A; see Fig. 1B for examples).

If Zld and Dl bind cooperatively and stabilize one another, one might expect that Dl may also help Zld binding. However, we found this not to be the case. When we carried out Zld ChIP-seq experiments in wild-type embryos and embryos that lack nuclear Dl (*gd*^*7*^) (Roth et al. 1989), we found only negligible effects on Zld binding; only 2.5% of the 10,376 Zld peaks identified (Fig. 1C), and 1.28% of the 2809 Dl-bound Zld peaks, showed significant differences between genotypes.

We next looked for salient features of the three Dl-peak groups. Group I Dl peaks, where Dl binding decreases in the absence of Zld, exhibit much stronger Zld binding than do peaks in the other two groups (Fig. 1D, see metaprofiles and Zld heatmap), suggesting that the effect of Zld on Dl binding is largely a local *cis*-regulatory effect rather than an indirect effect through, for instance, the misexpression of other TFs. A direct *cis*-regulatory relationship between Zld and Dl in Group I is also supported by the fact that regions around the Dl peak summits are highly enriched in both Dl and Zld motifs (Fig. 1D). Furthermore, a de novo motif search (see Methods) identified Zld and Dl motifs as the top two most enriched motifs in Group I regions (Supplemental Fig. 1A). In contrast, Group II and Group III peaks have substantially less Zld binding (Fig. 1D), Dl motifs are much less enriched, and Zld motifs are randomly distributed or even depleted around the Dl peak summits (Fig. 1D; Supplemental Fig. 1A).

Several lines of evidence suggest that Group I is highly enriched for developmental enhancers. First, analysis of the overall genomic distribution shows that, as Zld binding itself, Group I peaks mainly lie within intergenic and intronic regions, where enhancers typically reside (Supplemental Fig. 2A). Second, Group I peaks tend to be near genes that are zygotically expressed (Supplemental Fig. 2B). Finally, we found that Group I peaks overlap with many known enhancers, including known Dl targets such as *sna*, *sog*, and *brk* (e.g., Fig. 1B). However, Group I regions are also enriched for motifs of other TFs, such as Caudal, Bcd, Stat92E, Twi, and Trithorax-like (Trl; also known as GAGA factor or GAF) (Supplemental Fig. 1A), suggesting that Group I peaks may also include other patterning enhancers.

Since Dl binding is dependent on Zld in Group I, why can Dl bind in the absence of Zld to Group II and III regions? Group II and III peaks tend to be much more frequent near promoters (Fig. 1D, TSS column; Supplemental Fig. 2A). These promoters are highly enriched for Ohler1, Ohler6, and Ohler7 motifs (Ohler et al. 2002) and DNA-replication-related element (DRE) (Supplemental Fig. 1A; Hirose et al. 1993); and nearby genes tend to be continuously expressed maternally and zygotically (Supplemental Fig. 2B). This suggests that a large fraction of Group II and III regions are promoters of so-called housekeeping genes, which tend to have constitutively accessible promoters (Rach et al. 2009, 2011; Gaertner et al. 2012; Li and Gilmour 2013).

Group II and III peaks are also highly enriched for GAGA and the CTCF motif (Supplemental Fig. 1A), especially when regions near TSSs were eliminated before the de novo motif analysis (Supplemental Fig. 1B). GAF has known roles in opening chromatin (Tsukiyama et al. 1994; Okada and Hirose 1998) and is found at paused promoters (for review, see Gaertner and Zeitlinger 2014), Polycomb response elements (for review, see Müller and Kassis 2006; Schwartz and Pirrotta 2007), and Highly Occupied Target (HOT) regions where many TFs bind (Roy et al. 2010; Kvon et al. 2012; for review, see Slattery et al. 2012), all of which tend to be strongly depleted of nucleosomes and therefore may be accessible to Dl. Likewise, CTCF binding regions, which can be found near promoters, enhancers, and insulators are typically nucleosome-depleted (for review, see Phillips and Corces 2009; Ong and Corces 2014).

Thus, our analysis suggests that Dl may more generally redistribute to regions that are accessible in the genome in the absence of Zld, either because they are naturally accessible or because other factors such as GAF and CTCF may be able to open chromatin in the absence of Zld. Taken together, Group I are the potential target enhancers where Zld promotes Dl binding to a high level, thereby preventing ectopic Dl binding to other regions of the genome.

### Prominent nucleosome occupancy at Dl-bound regions in the absence of Zld

We next investigated how nucleosome occupancy is affected by Zld at the Dl group peaks and whether it dictates the binding changes we observed in *zld*^−^. We therefore performed MNase digestion with chromatin from 2–3 h wild-type and *zld*^−^ embryos followed by paired-end sequencing (see Methods).

**Figure 2.**
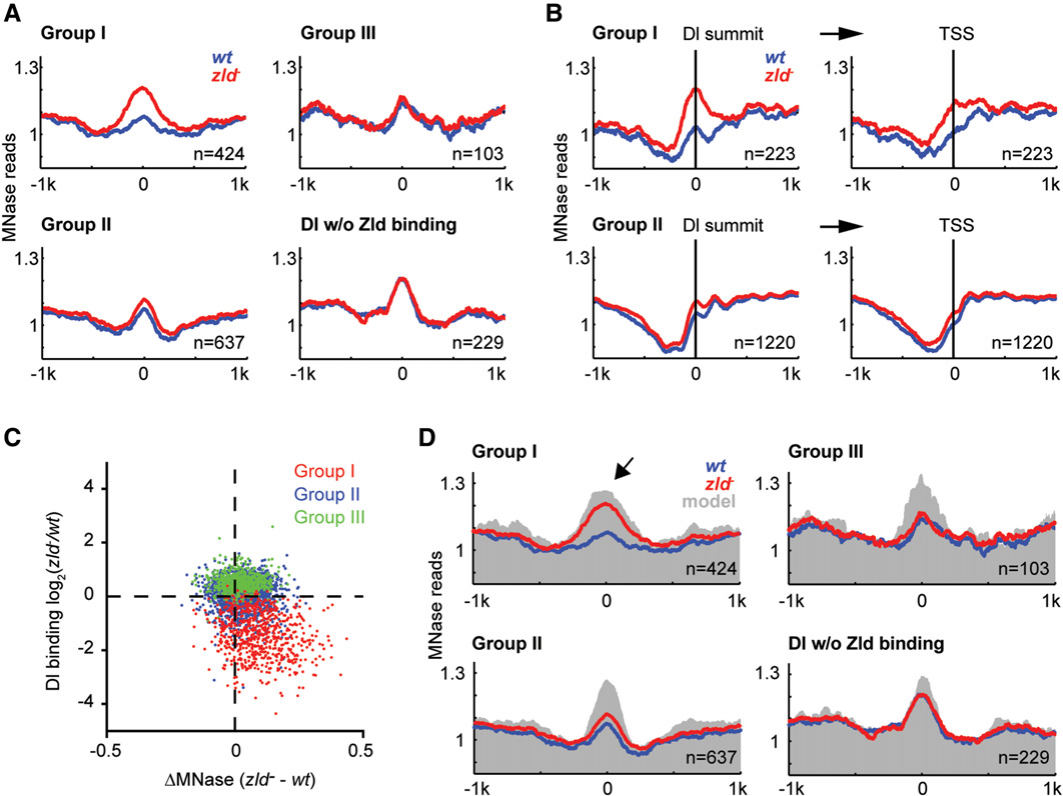
Loss of Dl binding in the absence of Zld is associated with increased nucleosome occupancy. Nucleosome occupancy was measured by MNase-seq (see Methods). (*A*) Metaprofiles (*wt* in blue, *zld*^−^ in red) of Zld-bound Dl peaks that are >1 kb away from a TSS are shown for the three Dl-peak groups, as well as Dl peaks that do not colocalize with Zld binding as control. The normalized MNase reads were aligned at the Dl summit, and average reads within 1 kb distance are shown. (*B*) Metaprofiles of Zld-bound Dl peaks that are ≤1 kb away from a TSS are shown for Groups I and II. The normalized MNase reads were either aligned at Dl summits (*left*) or the nearby TSSs (*right*). Note that the increased nucleosome occupancy in *zld*^−^ within Group I is much more pronounced on the *left*, arguing that the effect is directly due to Zld binding and not due to loss of transcription at these genes. (*C*) Scatter plot showing the correlation between ΔMNase (*x*-axis) and the fold change in Dl binding (*y*-axis) between *zld*^−^ and *wt* embryos. Values were calculated using the reads within 125 or 250 bp of the Dl summit for Dl binding and MNase, respectively. Note the strong correlation for Group I Dl peaks (red). (*D*) Metaprofiles of the Dl-peak groups as in *A*, but with the addition of the average predicted nucleosome occupancy based on the underlying DNA sequence (gray) using a published prediction model (Xi et al. 2010). Note that the high and broad nucleosome occupancy of Group I regions in *zld*^−^ is also predicted by the model (arrow), indicating that the role of Zld may be to tackle the intrinsically strong nucleosome barrier of 4–5 nucleosomes at these places, which would then help Dl access these regions.

Since transcription is altered in *zld*^−^ embryos (Liang et al. 2008; Nien et al. 2011) and transcription affects nucleosome occupancy, we initially analyzed Dl peaks that are not near a TSS (>1 kb). We also required these peaks to be bound by Zld and used the Dl peaks not bound by Zld as a control since they are unaffected by the loss of Zld (Supplemental Fig. 3). For all groups, we observed elevated central nucleosome occupancy around the Dl summit and a trough of lower occupancy at a 200–400 bp distance from the peak summit (Fig. 2A; Supplemental Fig. 4, heatmaps). However, for Group I, the central nucleosome occupancy was dramatically increased in *zld*^−^ embryos compared to wild-type (Fig. 2A). In contrast, the MNase profiles of Group II, Group III, and control peaks were indistinguishable between genotypes (Fig. 2A). This suggests that Zld plays an important role in reducing nucleosome occupancy at Group I target enhancers, and in the absence of Zld, these regions become highly occupied by nucleosomes.

We next asked whether the increased nucleosome occupancy was also visible at Dl peaks that are near a TSS (≤1 kb). This was indeed the case, and the pattern suggests that it is unlikely due to secondary effects from transcription (Fig. 2B). If this were the case, we would expect it to be most strongly visible when aligning the *zld*^−^ MNase profile at the TSS rather than the nearby Dl summit. However, the prominent nucleosome is lower after TSS alignment than after Dl peak summit alignment (Fig. 2B).

To quantify the difference in nucleosome occupancy between *zld*^−^ and wild-type embryos, we calculated the difference in MNase read counts within 250 bp of the Dl peak summit (ΔMNase) (see Methods). A positive score indicates a gain of nucleosome occupancy in *zld*^−^, i.e., loss of chromatin accessibility. As expected, we observed a significant negative correlation between the change in Dl binding and ΔMNase at Group I peaks (*r* = −0.19, *P* < 3.7 × 10^−7^) (Fig. 2C). In contrast, there was no correlation between Dl binding change and ΔMNase in Group II and III peaks. These results suggest that the high nucleosome occupancy in *zld*^−^ could prevent Dl from binding at high levels.

The increased nucleosome occupancy at Group I Dl peaks in the absence of Zld could be facilitated by nucleosome-favoring DNA sequences in these regions. This would be consistent with recent reports describing high intrinsic nucleosome occupancy at human enhancers (Tillo et al. 2010; Barozzi et al. 2014). We therefore used a published algorithm (Xi et al. 2010) to predict nucleosome occupancy in our Dl groups. All groups showed high predicted central nucleosome occupancy (gray shading in Fig. 2D) compared to random genomic regions of similar G-C content (Supplemental Fig. 5). Strikingly, the predicted nucleosome profile of Group I peaks shows an extended region of high nucleosome occupancy, similar to the actual MNase profile in *zld*^−^ embryos (Fig. 2D, arrow; compare gray shading and red line). This suggests that in the absence of Zld, nucleosome occupancy is at least in part dictated by intrinsic DNA sequence features.

The other Dl groups (Group II, III, and control peaks) also showed high predicted nucleosome occupancy, in fact, higher than the MNase data in both wild-type and *zld*^−^ embryos (gray area is higher than both red and blue lines in Fig. 2D). This suggests that Dl binding in these groups is goverened by other factors that reduce the high intrinsic nucleosome occupancy in these regions. Based on our motif analysis above (Supplemental Fig. 1B, including motif analysis of control peaks), GAF or CTCF are good candidates for explaining the difference between predicted and observed nucleosome occupancy in these groups.

### Early embryonic enhancers are characterized by Zld-dependent nucleosome depletion

**Figure 3.**
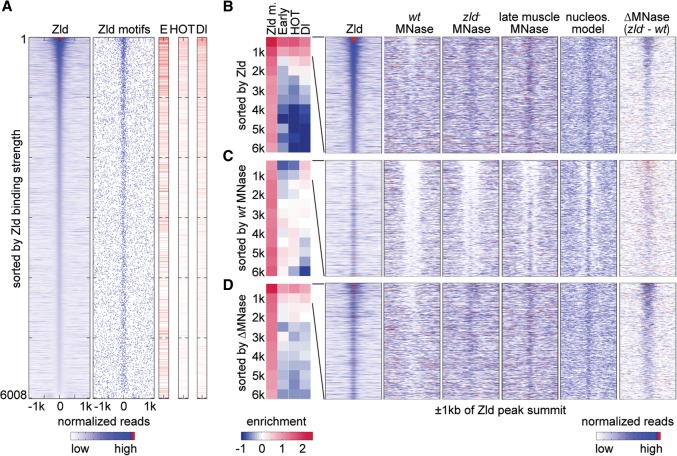
Zld binding and changes in nucleosome occupancy correlate with early enhancer activity. Analysis of 6008 Zld-bound regions that are >1 kb away from a TSS. In the heatmaps, normalized MNase-seq and ChIP-seq data for each region are aligned to the Zld summit, and 1-kb regions to each side are shown. (*A*) All 6008 non-TSS Zld-bound regions ranked by Zld summit reads from high to low show that Zld binding strongly correlates with Zld motifs and that the higher the Zld peak ranks, the more frequently early enhancers (E), HOT regions (HOT), and Dl peaks (Dl) overlap. The Zld peak rank also correlates with the degree of hotness, i.e., the number of TFs bound, and Dl binding strength, which are shown as degree of red in the same data. White indicates non-HOT regions. (*B*–*D*) The 6008 Zld-bound regions are ranked by Zld summit reads from high to low as in *A* (*B*); *wt* MNase (within 250 bp of Zld summits) from low to high (*C*), and ΔMNase (the read count difference between *zld*^−^ and *wt*) from high to low (*D*). The ranked data were then divided into bins of 500 regions (except for the last bin, which has 508 regions), and enrichment values for each bin are shown (blue for depletion and red for enrichment). The enrichment of Zld motifs (Zld m.) was calculated over genome background, while the enrichment of early enhancers (Early), HOT regions (HOT), and Dl peaks (Dl) was calculated over the average of the 6008 Zld-bound regions. Note that the enrichment at the top two bins is strongest when ranked by Zld summit reads, still strong when ranked by ΔMNase, and the lowest when ranked by *wt* MNase. Heatmaps for the top two bins (1000 regions) in each ranking is shown to the *right* for the following data: Zld binding, *wt* MNase, *zld*^−^ MNase, MNase profiles of 14–17 h muscle tissue (late muscle MNase), predicted nucleosome model (nucleos. model) (Xi et al. 2010), and ΔMNase. Note that the nucleosome occupancy in *zld*^−^ embryos resembles that of late muscle tissue, where Zld is also absent, as well as the predicted model, suggesting that in the absence of Zld, nucleosome occupancy is governed by DNA sequence features.

So far, we analyzed the dependence of Dl binding on Zld because the relationship between these two factors has been well studied (Nien et al. 2011; Kanodia et al. 2012; Foo et al. 2014). However, Zld also promotes the binding of other TFs (Yáñez-Cuna et al. 2012; Xu et al. 2014), raising the question of whether Zld promotes specific TF binding and enhancer activity more generally during ZGA through nucleosome depletion. We therefore aligned all non-TSS Zld-bound regions at the Zld peak summits and ranked them in order of Zld binding strength (reads at Zld peak summit) from high to low (*n* = 6008). As expected, Zld-bound regions were strongly enriched for Zld motifs, with the highest bound regions containing the most motifs (Fig. 3A).

We then analyzed how these Zld-bound regions overlap with known enhancers using a set of experimentally identified early enhancers derived from several databases, namely REDfly (Gallo et al. 2011), CRM Activity Database 2 (Bonn et al. 2012), and Fly Enhancers (Kvon et al. 2014). The data are visualized in Figure 3A, and a systematic enrichment analysis for each bin of 500 Zld peaks is shown in Figure 3B. The results show that the stronger Zld binds, the better the overlap with putative early enhancers (column “E” red lines in Fig. 3A), with the top two bins (top 1000 Zld-bound regions) being most enriched for early enhancers (column “Early” in Fig. 3B). This indicates that strong Zld binding is indeed a good marker for enhancer activity in the early embryo. Furthermore, Zld binding overlapped with HOT regions and Dl-bound regions (Fig. 3A,B), both of which are indicators of TF access. There was also a significant correlation between Zld binding levels and the number of TFs bound (*r* = 0.49, *P* < 1 × 10^−5^).

Focusing on the top 1000 Zld-bound regions, which have highest enhancer activity, we next examined the MNase profiles under different conditions. In wild-type embryos, these regions tend to have lower nucleosome occupancy, especially when Zld binding is the highest (*wt* MNase in Fig. 3B). In *zld*^−^ embryos, this trend is no longer visible, and nucleosome occupancy tends to be high across all Zld peaks (*zld*^−^ MNase in Fig. 3B).

Since the high intrinsic nucleosome occupancy at *Drosophila* enhancers is debated (Khoueiry et al. 2010; Kenigsberg and Tanay 2013), we tested this hypothesis further. To rule out that the high nucleosome occupancy is an artifact of the *zld*^−^ background, we also analyzed MNase from late embryo muscle tissue (Gaertner et al. 2012). Here, Zld is no longer expressed (Staudt et al. 2006; Liang et al. 2008), and early enhancers are expected to have ceased their activity. In this tissue, the Zld regions also have strikingly elevated nucleosome occupancy (late muscle MNase in Fig. 3B), consistent with the intrinsic disposition for nucleosomes in these DNA sequences (nucleos. model in Fig. 3B).

If the purpose of Zld is to deplete nucleosomes to make enhancers accessible, one might expect that low nucleosome occupancy in wild-type would correlate with enhancer activity. To test this, we ranked the Zld-bound regions (*n* = 6008) by wild-type MNase reads (±250 bp around Zld summit) from low to high and performed the same enrichment tests. We found that the most “open” regions in wild-type embryos are actually depleted of early enhancer activity and HOT regions (Fig. 3C), and there is little if any correlation between wild-type nucleosome occupancy and number of TFs bound (*r* = 0.01, *P* = 0.26), indicating that low nucleosome occupancy alone, as measured by MNase, is not a good proxy for TF binding and enhancer activity. Instead, we found that the low nucleosome occupancy in wild-type strongly correlates with the intrinsic nucleosome preference predicted by its underlying DNA sequences (*r* = 0.44, *P* < 1 × 10^−5^). Thus the most “open” regions tend to have nucleosome disfavoring sequences, especially in the regions flanking the Zld peak summits (nucleos. model in Fig. 3C). We conclude that such intrinsically “open” regions are unlikely to function as enhancers despite their apparent accessibility.

We therefore hypothesized that real enhancers are likely to be intrinsically “closed” and require Zld binding to become “open,” thus, the difference in nucleosome occupancy between *zld*^−^ and wild-type (ΔMNase) is predictive for enhancer function. In support of this, ΔMNase strongly correlates with Zld binding strength for the top 1000 Zld-bound regions (*r* = 0.54, *P* < 1 × 10^−5^) (ΔMNase in Fig. 3B), more so than *wt* MNase reads (*r* = −0.14, *P* < 1 × 10^−5^). Moreover, when we ranked 6008 Zld-bound regions by ΔMNase from high to low (Fig. 3D), we found that regions with the highest differences are strongly enriched for early enhancers as well as HOT regions and Dl-bound regions (Fig. 3D). Although the enrichment is not as high as that based on Zld ranking (Fig. 3B), it was much higher than that ranked by wild-type nucleosome occupancy (Fig. 3C). In addition, there is a significant correlation between ΔMNase and the number of TFs bound (*r* = 0.18, *P* = 5.6 × 10^−9^). This supports the idea that Zld-dependent changes in nucleosome occupancy, rather than low nucleosome occupancy per se, are important for enhancer activity. Taken together, our findings demonstrate that Zld binding and associated changes in nucleosome occupancy strongly correlate with early enhancer activity.

### The effect of Zld on nucleosome depletion is predominantly local

In our earlier analysis, we noticed that Zld-dependent Dl peaks (Group I, which includes many known early enhancers), have an extended region of high nucleosome occupancy (∼800 bp) when Zld is not present, whereas Group II and III peaks have a smaller region (∼400 bp) of nucleosome occupancy (see Fig. 2D). This raises the question whether early enhancers that are regulated by Zld generally have a disposition for an extended region of high nucleosome occupancy.

**Figure 4.**
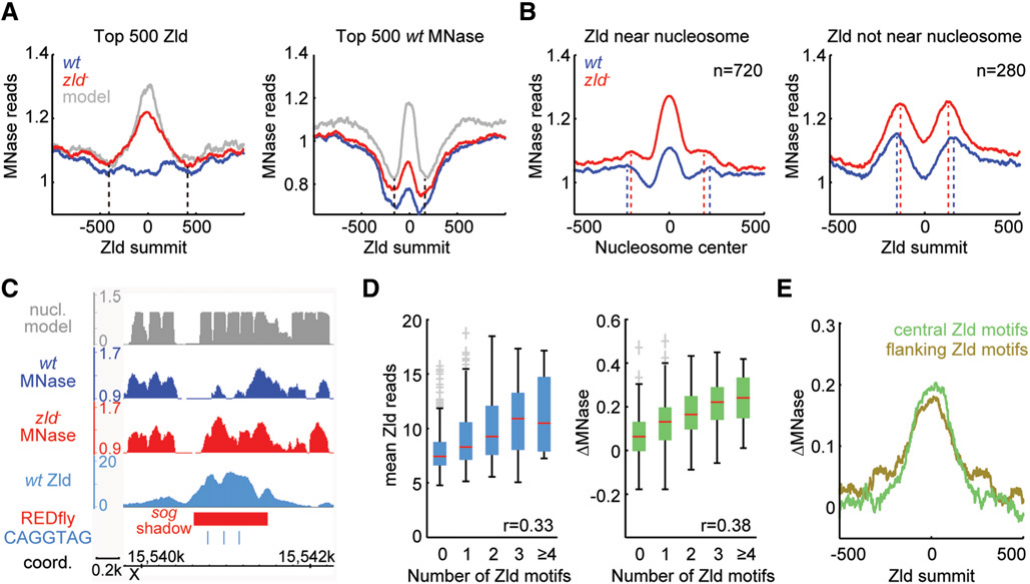
The effect of Zld on nucleosome occupancy is predominantly local. (*A*) Metaprofiles of nucleosome occupancy for the top 500 non-TSS Zld-bound regions as in Figure 3B, which are enriched for early enhancers (*left*), and the top 500 *wt* MNase regions (the most open regions) as in Figure 3C (*right*). For both plots, normalized MNase reads for *wt* (blue) and *zld*^−^ (red), as well as the predicted nucleosome occupancy (gray) (Xi et al. 2010), were aligned to the Zld summits. Note the difference in width of the region with high nucleosome occupancy (black vertical dashed lines), suggesting that early enhancers have extended regions of high nucleosome occupancy. (*B*) Nucleosome positions in *zld*^−^ MNase data were identified for the top 1000 non-TSS Zld-bound regions using the nucleR package (Flores and Orozco 2011). If the Zld summit mapped within 75 bp of a nucleosome center (*n* = 720), the normalized MNase data for *wt* (blue) and *zld*^−^ (red) were aligned at the center of that nearest nucleosome (*left*). For the remaining Zld-bound regions without a nucleosome within 75 bp (*n* = 280), the data were aligned at Zld summits (*right*). Note that in both plots, there is a notable shift for the nucleosome phasing between *wt* (blue) and *zld*^−^ (red), indicated by dashed lines. (*C*) IGB views showing predicted nucleosome model (gray track), *wt* MNase (blue track), *zld*^−^ MNase (red track), Zld binding (light blue track), REDfly enhancer (red rectangle), and CAGGTAG Zld motifs (light blue vertical lines) at the *sog* shadow enhancer region. (*D*) Box-and-whisker plots showing Zld binding (as mean ChIP reads; *left*) and ΔMNase (the read count difference between *zld*^−^ and *wt*; *right*) for the top 1000 non-TSS Zld peaks dependent on the number of Zld motifs within 125 bp of the Zld summits. The whiskers denote the interquartile range, and gray crosses mark outliers. Note that Zld binding and ΔMNase increase approximately linearly with increasing numbers of Zld motifs. (*E*) Metaprofiles of ΔMNase for regions with different configurations of Zld motifs among the top 1000 non-TSS Zld peaks. Regions with only 1 Zld motif within 125 bp of the Zld summit and at least one more Zld motif within 400 bp (flanking Zld motifs group; olive) have a wider profile than those with at least 2 Zld motifs confined within 125 bp of Zld summit and no more Zld motifs within 400 bp of the Zld summit (central Zld motifs group; green). Also note that the changes in nucleosome occupancy (ΔMNase) in the central Zld motif group, where the effect of Zld is confined, are strongest within 250 bp (1–2 nucleosomes) from the Zld summit, suggesting that Zld acts predominantly local.

Since early enhancer activity best correlated with Zld binding (see Fig. 3B), we selected the top bin of 500 Zld peaks and plotted the average nucleosome occupancy in wild-type and *zld*^−^ embryos as well as the predicted nucleosome occupancy based on DNA sequence. As a control, we analyzed the average nucleosome occupancy for the top 500 Zld peaks with maximum openness in wild-type embryos (Fig. 3C). Again, we found that regions with strong Zld binding tend to have broader regions of strong nucleosome occupancy than the control (Fig. 4A). Interestingly, this length (∼800 bp, theoretically covering ∼5 nucleosomes) is consistent with the reported size of early *Drosophila* enhancers (500–1000 bp) (Berman et al. 2004; Schroeder et al. 2004; Mirny 2010), suggesting that enhancers have higher nucleosome occupancy throughout their entire length.

A simple explanation for the extended nucleosome occupancy at regions with strong Zld binding would be that the Zld motifs themselves, which are typically found in multiple copies in these regions (Harrison et al. 2011; Nien et al. 2011), promote high nucleosome occupancy. However, when we altered the Zld motifs in these regions in silico (C changed to T, and G to A, which is known to disrupt Zld binding), the nucleosome prediction algorithm still predicted high nucleosome occupancy (Supplemental Fig. 6). This suggests that Zld motifs per se are not sufficient to promote high nucleosome occupancy in the absence of Zld and is consistent with previous reports that additional sequences outside TF binding motifs promote nucleosome occupancy (Kenigsberg and Tanay 2013; Barozzi et al. 2014). We therefore propose that the high intrinsic nucleosome occupancy of *Drosophila* early embryonic enhancers is an inherent feature of the enhancers’ function as has been reported for human enhancers (Tillo et al. 2010; Barozzi et al. 2014).

The length of enhancers and high nucleosome occupancy implies that the access to enhancers is frequently controlled by multiple nucleosomes. This in turn raises the question of whether Zld binding can trigger the removal of multiple nucleosomes or whether the effect of Zld is locally restricted. Furthermore, it raises the question of whether Zld binding affects the positioning of nucleosomes.

Average MNase profiles aligned to the Zld peak summit do not show clear nucleosome phasing (Fig. 4A). In order to visualize nucleosome positioning, we used a published algorithm to identify the nearest nucleosome to each Zld summit (Flores and Orozco 2011). The regions where the nucleosome center was found within 75 bp of the Zld peak summit (72% of the top 1000 Zld peaks) were then aligned at the nucleosome center (Fig. 4B, left). The average MNase pattern indicates that Zld replaces the aligned nucleosome upon binding in wild-type, and this effect is predominantly local, although there is also a more long-range effect. At the remaining 28% Zld-bound regions, where the Zld summit is >75 bp away from the closest nucleosome center, the change in MNase between wild-type and *zld*^−^ is now observed at the two flanking nucleosomes (Fig. 4B, right). This suggests that Zld does not necessarily need to bind close to the nucleosome center to displace nucleosomes, but its effect is nevertheless strongest locally (1–2 nucleosomes from the peak summits).

Interestingly, we observed that the nucleosomes flanking the Zld peaks are slightly further apart in wild-type compared to *zld*^−^ (∼50 bp in each case) (Fig. 4B), raising the possibility that Zld binding can affect nucleosome positioning. It is unclear whether this could be a direct effect of the Zld protein, which is large (∼180 kDa) (Nien et al. 2011), or whether Zld may affect nucleosome phasing indirectly through other TFs or chromatin remodeling.

If Zld mostly affects 1–2 nucleosomes but early enhancers may extend over ∼5 nucleosomes, the implication is that depleting multiple nucleosomes may be accomplished by multiple Zld motifs within an enhancer. Indeed, we found that known early enhancers with multiple Zld motifs often have extended regions of nucleosome depletion (see *sog* enhancer in Fig. 4C).

We therefore analyzed the effect of increasing numbers of Zld motifs on ΔMNase. We found that the more Zld motifs within 125 bp centered on the Zld summit, the higher the Zld binding and the higher the magnitude of ΔMNase (Fig. 4D), supporting the idea that multiple Zld motifs have a stronger effect on depleting nucleosomes in enhancers.

Finally, we asked whether the location of multiple Zld motifs within enhancer regions may affect the pattern of nucleosome depletion. For this, we compared the regions with at least two Zld motifs within 125 bp but none in the flanking nucleosomes (central Zld motifs group) with those regions bearing Zld motifs in the flanking regions but only one central Zld motif (flanking Zld motifs group). Although both groups have a similar amount of total Zld motifs, their ΔMNase pattern was slightly different. For the central Zld motifs group, the Zld-dependent changes in MNase are more concentrated at the center, whereas they extend further out for regions with flanking Zld motifs (Fig. 4E). This further supports the hypothesis that Zld's strongest effect is local and that the number and position of Zld motifs have an effect on the pattern of nucleosome depletion.

## Discussion

### Zld counteracts a strong intrinsic nucleosome barrier

An important finding from our study is that early enhancers acquire high nucleosome occupancy about the length of typical enhancers in the absence of Zld. These regions have high predicted nucleosome occupancy based on underlying DNA sequences and acquire high nucleosome occupancy in wild-type embryonic tissues when Zld is no longer present during late embryogenesis. Taken together, these data show that early enhancers generally have a strong intrinsic nucleosome barrier.

Previous evidence on the intrinsic nucleosome occupancy at enhancers has been conflicting since it has been reported as either low (Daenen et al. 2008; Khoueiry et al. 2010) or high (Tillo et al. 2010; Barozzi et al. 2014). Our results unambiguously demonstrate high intrinsic nucleosome occupancy at early *Drosophila* enhancers, since we not only predict intrinsic nucleosome occupancy but also demonstrate high nucleosome occupancy experimentally (as observed in *zld*^−^ embryos and in wild-type late muscle tissue). This has important implications for the well-studied function of early *Drosophila* enhancers.

The simplest model is that the high nucleosome occupancy in the absence of appropriate TFs protects enhancers from inappropriate binding and activation. However, a more intriguing model proposed by Mirny (2010) poses that high nucleosome occupancy promotes a specific type of TF cooperativity called cooperative nucleosome binding (Adams and Workman 1995). Experimental evidence showed that TFs can dramatically enhance each others’ binding to nucleosomal DNA simply by competing against a common nucleosome (Adams and Workman 1995). Thus, the higher the nucleosome barrier, the more TFs are required to break the histone–DNA contacts. This in turn makes the enhancer activity dependent on multiple TFs without requiring direct physical interactions between them. This model fits well for our system since early *Drosophila* enhancers are strongly controlled by the combinatorial input of multiple TFs (for review, see Spitz and Furlong 2012; Shlyueva et al. 2014), and no strict motif grammar has been found between their binding motifs (for review, see Arnosti and Kulkarni 2005; Lusk and Eisen 2010).

Since our ChIP results show that Dl binding depends on Zld, but not the other way around, there is a hierarchy by which TFs activate enhancers in a combinatorial manner. We propose that Zld's pioneering role is its ability to lower (or prevent) the very high nucleosome barrier in each enhancer, and it does so just enough to allow patterning TFs to bind and to help antagonize the remaining nucleosome barrier. Such partial nucleosome depletion by Zld is supported by our findings that binding of Zld only leads to a relatively local depletion of about 1–2 nucleosomes within an enhancer, that multiple Zld binding motifs lead to stronger depletion, and that the position of the Zld motifs within the enhancer matters. The degree of nucleosome depletion by Zld thereby sets a threshold required for patterning TFs such as Dl to achieve robust transcriptional activation, consistent with our recent observations (Foo et al. 2014).

It should be noted that the mechanism by which Zld induces nucleosome depletion remains unknown. In the simplest scenario, Zld might bind to its targets very early during the rapid nuclear cycles, when the chromatin may not be as densely packed and thus more accessible (Lowenhaupt et al. 1983; Harrison et al. 2011; Li et al. 2014), and then prevent nucleosomes from being assembled nearby. Alternatively, Zld may bind, destabilize, and eject nucleosomes, thereby acting as a more classical pioneer factor (for review, see Zaret and Carroll 2011; Iwafuchi-Doi and Zaret 2014). Regardless of whether Zld can bind its motifs embedded in nucleosomes, Zld's ability to reduce nucleosome occupancy and facilitate the binding of TFs certainly fulfills a pioneering role.

The pioneering role we presented here for Zld during *Drosophila* ZGA may be a general feature of key zygotic genome activators. For example, Pou5f3, which controls ZGA together with Nanog and SoxB1 family proteins in zebrafish (Lee et al. 2013; Leichsenring et al. 2013) also binds before ZGA. Interestingly, the mammalian homolog of Pou5f3, Pou5f1 (also known as Oct4), is a pluripotency factor that, along with Sox2 and Klf4, gains initial access to closed chromatin at enhancers of genes promoting reprogramming from fibroblasts to induced pluripotent stem cells (Soufi et al. 2012; for review, see Soufi 2014). This points to a mechanistic link between ZGA and cellular reprogramming, the center of which may be the pioneering activity to potentiate TF binding and gene expression as exemplified by Zld.

### A model for how Zld primes enhancers during ZGA

**Figure 5.**
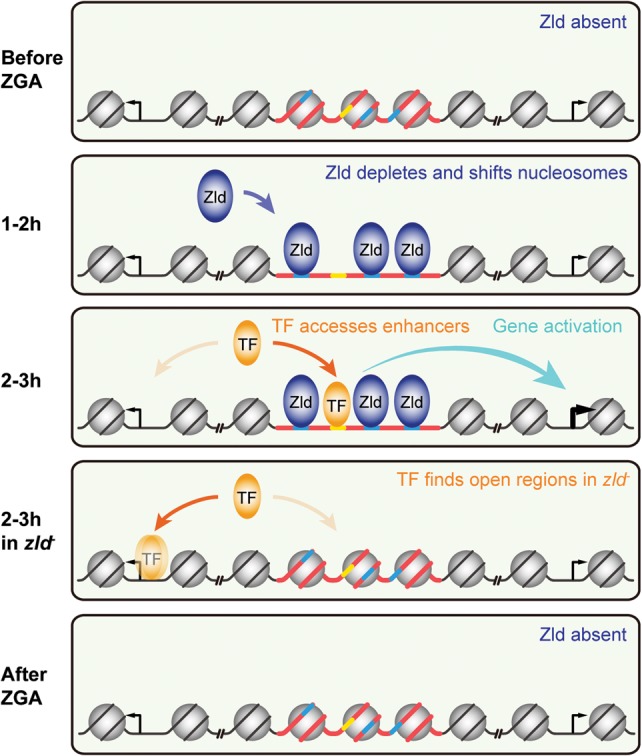
Model of Zld's role on TF specific binding and enhancer activation. When Zld is absent before ZGA, enhancers (red bold line) bearing Zld motifs (blue bold line) and other TF motifs (yellow bold line) are covered by nucleosomes (gray circle) due to intrinsic nucleosomal preference (as far as nucleosome formation can occur during the rapid nuclear division cycles). Once Zld is present (dark blue oval), Zld binding to its motifs leads to local nucleosome depletion and possibly nucleosome shifting, which exposes the motifs of other TFs within the same enhancer. Patterning TFs (orange oval) can now access their motifs, which may lead to enhancer activation (cyan arrow). In *zld*^−^ embryos, TFs are occluded from binding due to the high intrinsic nucleosome barrier at enhancers. Instead, excess TFs now bind nonspecifically (fuzzy orange oval) to open regions without cognate motifs, such as promoters. As development proceeds and Zld protein levels diminish in most late embryonic tissues, Zld decommissions from binding and TF motifs are again occluded from TF access.

Taken together, we propose the following temporal working model for how Zld primes early embryonic enhancers during ZGA (Fig. 5). As the Zld protein level rises in the first hour of development (Harrison et al. 2010; Nien et al. 2011), Zld begins to locally reduce nucleosome occupancy at target enhancers that normally have a high intrinsic nucleosome barrier. This is unlikely to be solely an effect of histone acetylation, which accompanies early Zld binding, since acetylated histones are more broadly found over Zld-bound regions (Li et al. 2014).

Starting 1–2 h and peaking at 2–3 h, patterning TFs such as Dl gain access to these enhancers. In certain embryonic regions, where the right combination of patterning TFs is present, Zld and these TFs then strongly bind through collaborative nucleosome binding and activate transcription in a distinct pattern in the embryo. In this process, some TFs such as Dl might be more strongly dependent on prior chromatin accessibility. A recent genome-wide analysis identified NFκB family members, mammalian homologs of Dl, as “settler” TFs whose binding is strongly governed by the accessible chromatin created by “pioneer” TFs (Sherwood et al. 2014).

In the absence of Zld, binding of Dl is severely diminished. This is accompanied by a redistribution of Dl to other regions in the genome that remain accessible. Such TF redistribution in the absence of a key activator has been observed previously in yeast (Zeitlinger et al. 2003), flies (Xu et al. 2014), and mammalian systems (Sahu et al. 2011; Wang et al. 2011; Theodorou et al. 2013). The simplest explanation for this phenomenon is the law of mass action, i.e., given that the nuclear Dl concentration remains the same, more unbound Dl is available to drive ectopic binding. A good candidate for facilitating ectopic Dl binding in the absence of Zld is GAF, since we found the ectopic Dl-bound regions to be enriched for the GAGA motif.

When this early pattern formation phase ends and Zld levels begin to decrease (Kanodia et al. 2012), the nucleosome-favoring sequences promote high nucleosome occupancy at these regions, closing enhancers and reducing transcriptional output. Thus, Zld acts as a timer of ZGA in that it controls the engagement and decommission of TFs at target enhancers by transiently reducing the nucleosome barrier. Since the Zld-mediated nucleosome depletion strongly correlates with early enhancer activity, it is likely a central mechanism by which Zld specifies and primes enhancers across the genome. It will be interesting to analyze whether this is a general property of zygotic genome activators and whether other pioneer factors play similar roles at later stages of development.

## Methods

### Fly strains

*Orego-R* was used as wild-type (*wt*) strain. *gd*^*7*^/*gd*^*7*^ and *gd^7^/Y* flies were obtained from *gd^7^/winscy, P{hs-hid}5* parents, which were heat-shocked during the second to third instar larval stage at 37°C for 1 h for 2 d. *zld*^−^ embryos were depleted of maternal Zld through the “Maternal-Gal4-shRNA” system (Staller et al. 2013), where *MTD-Gal4/UAS-shRNA-zld* females were crossed to *wt* males. The *MTD-Gal4* stock (Petrella et al. 2007), which drives robust Gal4 expression throughout oogenesis, as well as the passenger strand sequence CGGATGCAAGTTGCAGTGCAA targeting *zld* transcripts (shRNA-*zld*) were obtained from the Perrimon laboratory. The UAS-shRNA*-zld* vector was then constructed using the Valium22 vector and injected as previously described (Ni et al. 2011). Maternal *zld*^−^ embryonic phenotypes, as described in Liang et al. (2008) using a *zld* null allele in germ line clones, were confirmed by embryonic lethality, in situ hybridization of Zld target genes, and immunofluorescent staining with antibodies against Zld (Nien et al. 2011; data not shown).

### Embryo collection, fixation, and sorting

After preclearing, 2–3 h *wt*, *gd*^*7*^, or *zld*^−^ embryos were collected, dechorionated, and fixed with 1.8% formaldehyde for 15 min as described (Chen et al. 2013). For sorting, fixed embryos were rehydrated with PBT and stained with DAPI. Post-nc14 and disorganized embryos were removed under a Leica DM IL inverted microscope as described (Chen et al. 2013).

### ChIP-seq

Two biological replicates were performed for each ChIP experiment as described (Chen et al. 2013). Extracted chromatin was incubated with sheep anti-Rabbit IgG Dynabeads (Life Technologies) coated with antibodies against Zld (Nien et al. 2011) or Dl (He et al. 2015). Libraries were prepared from ChIP and input DNA using the NEBNext DNA Library Prep Master Mix Set for Illumina kit either following the manufacturer's instructions, or with a modified protocol (Chen et al. 2013), and single-end sequenced on an Illumina HiSeq 2000. ChIP and input sequencing reads and coverage are detailed in Supplemental Figure 7.

### MNase-seq

Two biological replicates were performed for each MNase digestion experiment as described (Chen et al. 2013). Briefly, chromatin was extracted from 100–150 µL of sorted 2–3 h *wt* and *zld*^−^ embryos per replicate, then digested with an MNase (Worthington Biochemical Corporation #LS004798) concentration gradient of 20, 10, 5, 5/2, 5/4, 5/8, 5/16, 5/32, and 0 units (negative control) for 30 min at 37°C. Mononucleosome-sized DNA was extracted from lanes containing 20 and 10 units MNase digested samples on a 1.7% agarose gel, when the dinucleosome-sized DNA band just disappeared, indicating saturation but not overdigestion. Libraries were prepared using the NEBNext DNA Library Prep Master Mix Set for Illumina kit either following the manufacturer's instructions, or with a modified protocol (Chen et al. 2013), then subjected to paired-end sequencing on an Illumina HiSeq 2500 sequencing system. A typical nucleosome phasing pattern at TSSs was observed similar to that shown in Figure 4C of Chen et al. (2013) for 2–3 h embryos, confirming that the MNase-seq data can resolve nucleosome footprints (data not shown). MNase sequencing reads and coverage are detailed in Supplemental Figure 7.

### Sequence alignment and normalization

All sequencing reads were aligned to the *Drosophila melanogaster* genome (dm3, BDGP Release 5) using Bowtie (v0.12.7) (Langmead et al. 2009), allowing a maximum of two mismatches and including only uniquely aligned reads. Aligned reads were then extended to the average insert size of the library estimated by Bioanalyzer for ChIP-seq or extended to the corresponding paired-end tag for MNase-seq. Extended MNase reads were then log_2_ transformed and *Z*-score transformed for normalization (Rizzo et al. 2012). After normalization, regions flanking the peak summits are at similar levels in different genotypes (Figs. 2A,D, 4A) and replicates (data not shown).

### ChIP peak finding and normalization

Unextended uniquely mapped reads from the two Dl replicates (*R* = 0.85), or two Zld replicates (*R* = 0.85), respectively, were combined using SAMtools (Li et al. 2009). MACS (v1.4) (Zhang et al. 2008) was used to call peaks with the default setting and the “call-subpeaks” function. Peaks were also called from each replicate with the same setting. Only peaks from the combined set that also existed in both replicates were defined as “combined peaks” and used for subsequent analyses.

To normalize ChIP and input reads, we combined extended uniquely mapped reads from two replicates, divided by genome-wide median coverage, and performed *Z*-score transformations using the mean and standard deviation of mapped reads outside the “combined peak” regions, giving rise to “normalized reads.” “Combined peaks” that mapped to Chromosomes U and Uextra, or peaks where the normalized ChIP reads were less than the normalized input reads within 100 bp of the peak summit (or the entire region if <200 bp) were discarded. The remaining peaks were termed “enriched combined peaks.”

In order to compare differential Dl binding between *wt* and *zld*^−^, or differential Zld binding between *wt* and *gd*^*7*^, the union of “enriched combined peaks” from both genotypes was analyzed so that the exact same regions were under comparison, hereafter referred to as “DESeq regions” (see DESeq analysis below). For Dl ChIP analyses, Dl DESeq regions from the combined set were called “Dl-bound regions.”

For Zld ChIP analyses other than DESeq, Zld replicate 1 was used for MACS peak calling as described above. Log_2_(ChIP/input) was obtained and *Z*-score transformed using the mean and standard deviation of mapped reads outside Zld peaks, with a cutoff of *Z*-score ≥ 1.6445 (*P* < 0.05). Peaks mapping to Chromosomes U and Uextra were excluded from further analyses. The remaining peaks were defined as “Zld-bound regions.”

### Differential binding analysis by DESeq

To examine differential Dl binding between *wt* and *zld*^−^, normalized *wt* and *zld*^−^ ChIP and input reads from each DESeq region of two replicates were processed with the DESeq package, with the default locfit package setting (Anders and Huber 2010). Dl-bound regions in *zld*^−^ were considered significantly decreased if the DESeq region had log_2_(*zld*^−^/*wt*) < 0 and FDR < 0.1, and significantly increased if log_2_(*zld*^−^/*wt*) > 0 and FDR < 0.1, or labeled as unchanged if FDR ≥ 0.1. The differential Zld binding analysis between *wt* and *gd*^*7*^ was performed in the same manner. To rule out the possibility that the phenomenon we saw was a normalization bias caused by the DESeq package, we independently calculated the differences of normalized ChIP reads within 125 bp of Dl summits (or the entire region if <250 bp) from the combined set for each Dl DESeq region between *wt* and *zld*^−^, or the differences of normalized ChIP reads within 125 bp of Zld summits (or the entire region if <250 bp) from the combined set for each Zld DESeq region between *wt* and *gd*^*7*^, which verified the DESeq results (data not shown).

We also controlled for our Z-score normalization method by normalizing with the mean of total reads, which yielded very similar properties of the Dl-bound regions (DESeq and MNase profiles shown in Supplemental Fig. 8).

### Determination of overlapping regions

Two regions were considered overlapping if there was at least 1 bp overlap.

### Zld and Dl motif analysis

Eight Zld motifs (Nien et al. 2011) were used for Zld motif analysis. GGGWWWWCYS GGGWDWWWCYS GGGWWWWCCM and GGGDWDWWWCCM were used for Dl motif analysis. In Figure 3, within each nonoverlapping bin of non-TSS Zld peaks, Zld motif enrichment is the average density of Zld motifs within 250 bp of peak summits (or the entire bound region if <500 bp) divided by the density of Zld motifs within the *Drosophila* genome.

### HOMER de novo motif discovery

De novo motif discovery was performed using the HOMER software (v4.5) (Heinz et al. 2010) with the default setting. Motifs were searched for within 100 bp of Dl peak summits (or the entire bound region if <200 bp) from the combined set.

### Nucleosome model prediction

We used NuPoP (R package) (Xi et al. 2010) to predict the probability of nucleosome occupancy over the entire *Drosophila melanogaster* genome (dm3, BDGP Release 5).

### Comparing changes in nucleosome occupancy between *wt* and *zld*^−^ embryos

ΔMNase in Figures 2C and 3B–D was calculated as the difference between normalized MNase reads within 250 bp of the Dl (or Zld) peak summit from *zld*^−^ versus *wt*, excluding regions with 0 reads from the averaging process. The same was true for Figure 4D, except that it was calculated within 125 bp of the Zld peak summit.

### Enhancer collection and assignment

Early enhancers were collected from REDfly enhancers (Gallo et al. 2011) that are active minimal CRMs with the expression term “blastoderm embryo” (stages 3–5), CRM Activity Database 2 enhancers (Bonn et al. 2012) active during stages 5–6, and Fly Enhancers (Kvon et al. 2014) active during stages 4–6, excluding those with the exact same region.

### Enrichment of enhancers, HOT regions, and Dl peaks in Zld-bound regions

HOT regions were defined as hotness ≥8 (Roy et al. 2010). Non-TSS Zld peaks have boundaries >1 kb away from a TSS. Enhancers, HOT regions, and Dl peaks were considered as bound by non-TSS Zld if they had 1 bp overlap. In Figure 3, for each bin of non-TSS Zld peaks, the enrichment was calculated as the observed number of overlapping regions divided by the expected number (average for all bins).

### The number of TFs overlapping with Zld peaks

As a measurement for the number of bound TFs related to Figure 3, the hotness score (Roy et al. 2010) was used. If multiple TF-bound regions overlapped with one Zld peak, the highest hotness score was used.

### Analysis of nucleosome positioning

We used nucleR (R package) (Flores and Orozco 2011) to analyze the nucleosome positioning of MNase data sets in Figure 4B. The mapped and extended MNase-seq data sets were filtered by Fourier filter in nucleR package, followed by nucleosome calling with the default setting.

## Data access

All ChIP-seq and MNase-seq data from this study have been submitted to the NCBI Gene Expression Omnibus (GEO; http://www.ncbi.nlm.nih.gov/geo/) under accession number GSE65441.

## Supplementary Material

Supplemental Material
