# Supplementary material for: Zelda overcomes the high intrinsic nucleosome barrier at enhancers during Drosophila zygotic genome activation
Source: Genome Res. 2015 Nov;25(11):1703–14. doi: 10.1101/gr.192542.115 (PMC4617966; doi:10.1101/gr.192542.115)
Supplement: Supplemental Material [file supp_25_11_1703__index.html]

Zelda overcomes the high intrinsic nucleosome barrier at enhancers during Drosophila zygotic genome activation — Zelda overcomes the high intrinsic nucleosome barrier at enhancers during Drosophila zygotic genome activation — Supplemental Material 

# Zelda overcomes the high intrinsic nucleosome barrier at enhancers during *Drosophila* zygotic genome activation

## Supplemental Material

**Files in this Data Supplement:**

- Supplemental Figures.pdf
