# Supplementary material for: Zelda overcomes the high intrinsic nucleosome barrier at enhancers during Drosophila zygotic genome activation
Source: Genome Res. 2015 Nov;25(11):1703–14. doi: 10.1101/gr.192542.115 (PMC4617966; doi:10.1101/gr.192542.115)
Supplement: Supplemental Material [file supp_gr.192542.115_Supplemental_Figures.pdf]

Sun_Supplemental Fig1A

Group I

rank	HOMER *de novo* motif	p-value	similar motif	% of targets	% of background	enrichment
1	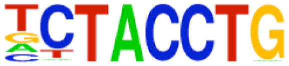	1E-208	Zelda	41.97%	3.92%	10.71
2	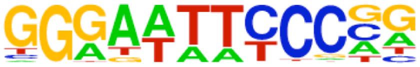	1E-47	Dorsal	13.55%	1.88%	7.21
3	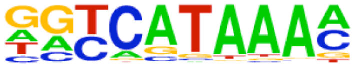	1E-45	Caudal	19.59%	4.47%	4.38
4	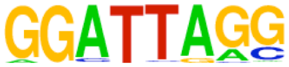	1E-19	Bicoid	20.47%	8.96%	2.28
5	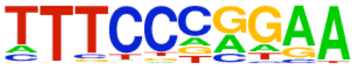	1E-18	STAT92E	20.03%	8.81%	2.27
6	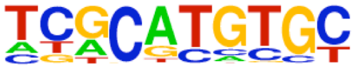	1E-15	Twist	11.19%	3.87%	2.89
7	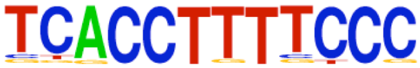	1E-14		1.77%	0.05%	35.40
8	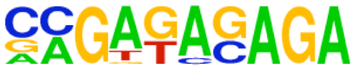	1E-14	GAGA	11.49%	4.29%	2.68
9	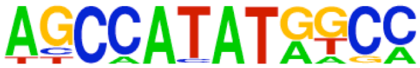	1E-12	Pho	1.77%	0.07%	25.29
10	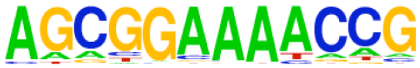	1E-12	Dorsal	5.45%	1.28%	4.26

Sun_Supplemental Fig1A, cont.

Group II

rank	HOMER *de novo* motif	p-value	similar motif	% of targets	% of background	enrichment
1	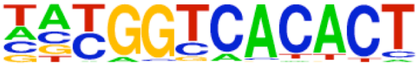	1E-113	Ohler1	11.90%	2.00%	5.95
2	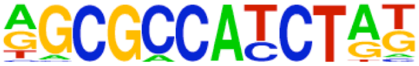	1E-95	CTCF	4.96%	0.26%	19.08
3	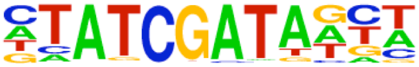	1E-74	DRE	10.11%	2.24%	4.51
4	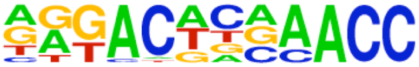	1E-51	matches DPE	3.95%	0.43%	9.19
5	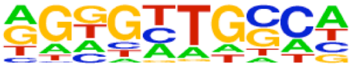	1E-48	Ohler8	9.97%	3.13%	3.19
6	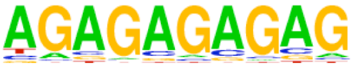	1E-47	GAGA	17.65%	7.97%	2.21
7	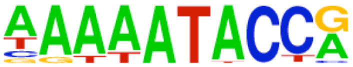	1E-33	Ohler6	5.84%	1.61%	3.63
8	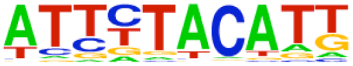	1E-30	CG11617	5.06%	1.34%	3.78
9	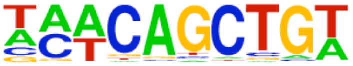	1E-25	Ohler5	5.42%	1.72%	3.15
10	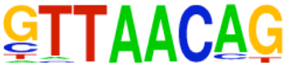	1E-25	Ovo	8.69%	3.72%	2.34
11	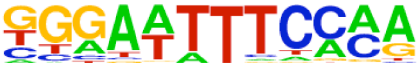	1E-22	Dorsal	18.01%	10.82%	1.66
12	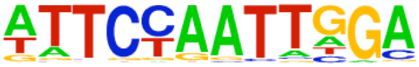	1E-22	Invected	4.27%	1.25%	3.42
13	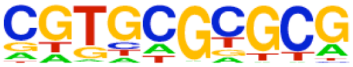	1E-20	CG-repeat	13.60%	7.68%	1.77
14	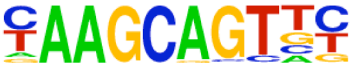	1E-17	Grainy head	2.16%	0.44%	4.91
15	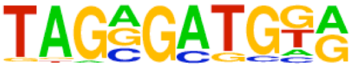	1E-17	Ohler7	2.76%	0.71%	3.89
16	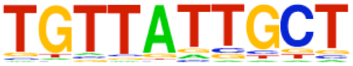	1E-17	Caupolican	20.22%	13.59%	1.49

Sun_Supplemental Fig1A, cont.

Group III

rank	HOMER *de novo* motif	p-value	similar motif	% of targets	% of background	enrichment
1	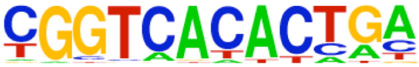	1E-58	Ohler1	12.42%	1.02%	12.18
2	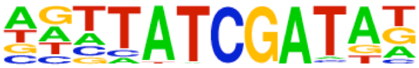	1E-36	DRE	13.35%	2.37%	5.63
3	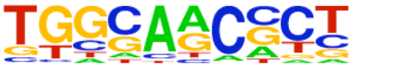	1E-35	matches MTE	11.18%	1.64%	6.82
4	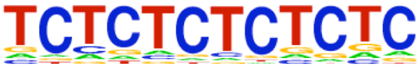	1E-26	GAGA	21.43%	7.80%	2.75
5	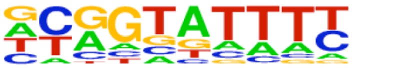	1E-20	Ohler6	9.94%	2.41%	4.12
6	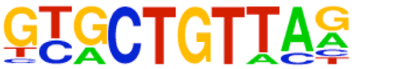	1E-19	Ovo	6.52%	1.06%	6.15
7	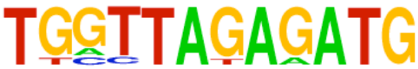	1E-19	Ohler7	1.24%	0.00%	N/A
8	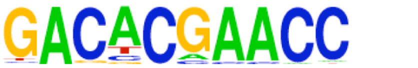	1E-19	Deaf1	4.66%	0.48%	9.71
9	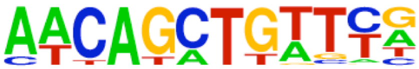	1E-18	Ohler5	7.76%	1.64%	4.73
10	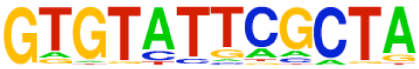	1E-16	Slp1	5.28%	0.81%	6.52
11	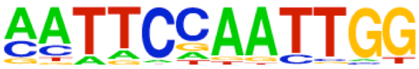	1E-15		6.99%	1.55%	4.51
12	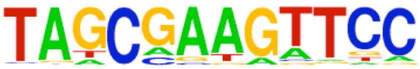	1E-14	Hsf	3.42%	0.35%	9.77
13	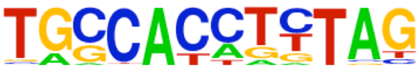	1E-13	Twist	4.04%	0.56%	7.21
14	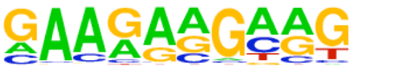	1E-12		7.76%	2.37%	3.27
15	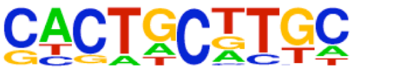	1E-12		4.04%	0.67%	6.03

Sun_Supplemental Fig1B

Group I (non-TSS, w/ Zld binding)

rank	HOMER *de novo* motif	p-value	similar motif	% of targets	% of background	enrichment
1	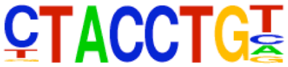	1E-125	Zelda	41.98%	4.19%	10.02
2	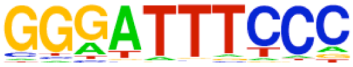	1E-37	Dorsal	28.07%	7.28%	3.86
3	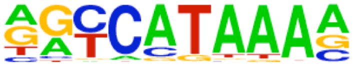	1E-28	Caudal	21.70%	5.65%	3.84
4	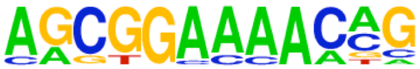	1E-14	Dorsal	4.01%	0.26%	15.42
5	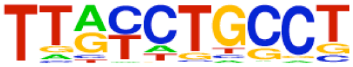	1E-14	Zelda	20.99%	8.71%	2.41
6	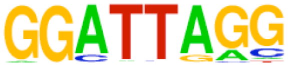	1E-12	Bicoid	15.33%	5.68%	2.70

Group II (non-TSS, w/ Zld binding)

rank	HOMER *de novo* motif	p-value	similar motif	% of targets	% of background	enrichment
1	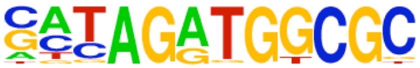	1E-59	CTCF	9.58%	0.43%	22.28
2	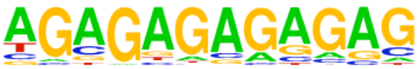	1E-25	GAGA	23.55%	9.43%	2.50
3	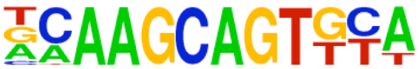	1E-23	Grainy head	4.24%	0.26%	16.31
4	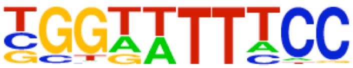	1E-21	Dorsal	9.42%	1.99%	4.73
5	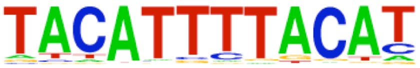	1E-18	CG11617	6.59%	1.15%	5.73
6	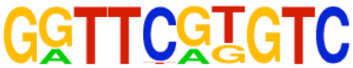	1E-16	Deaf1	3.14%	0.21%	14.95
7	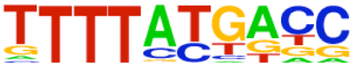	1E-16	Caudal	14.29%	5.39%	2.65
8	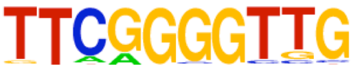	1E-15	Deaf1	2.98%	0.21%	14.19
9	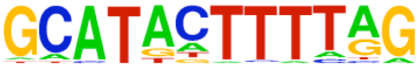	1E-15	su(Hw)	4.40%	0.60%	7.33
10	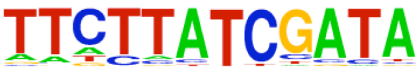	1E-13	DRE	5.18%	0.97%	5.34

Sun_Supplemental Fig1B, cont.

Group III (non-TSS, w/ Zld binding)

rank	HOMER *de novo* motif	p-value	similar motif	% of targets	% of background	enrichment
1	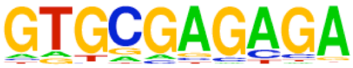	1E-17	GAGA	47.57%	12.18%	3.91
2	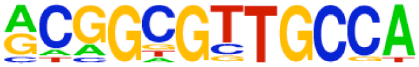	1E-14	Ohler8	14.56%	0.81%	17.98

Control (non-TSS, Dl w/o Zld binding)

rank	HOMER *de novo* motif	p-value	similar motif	% of targets	% of background	enrichment
1	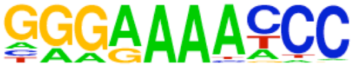	1E-15	Dorsal	9.17%	0.80%	11.46
2	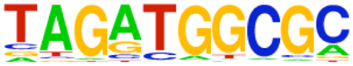	1E-13	CTCF	16.59%	3.83%	4.33

Supplemental Fig. 1. HOMER *de novo* motif discovery. *De novo* motifs found in Group I, II, III Dl peak regions are shown for (**A**) all Dl peaks, and (**B**) Dl peaks >1kb away from a TSS that are bound or unbound by Zld, ranked by enrichment p-value. Possible false positive *de novo* motifs are not shown; only top 16 out of 21 motifs are shown for Group II peaks in (A), and only top 10 out of 13 motifs are shown for Zld bound non-TSS Dl Group II peaks in (B). The name of similar motif, or the protein bound to the similar motif, is listed if known. The percentage of the *de novo* motif identified in each Dl peak group and from background is shown, together with motif enrichment over background (% of targets/% of background).

Sun_Supplemental Fig2

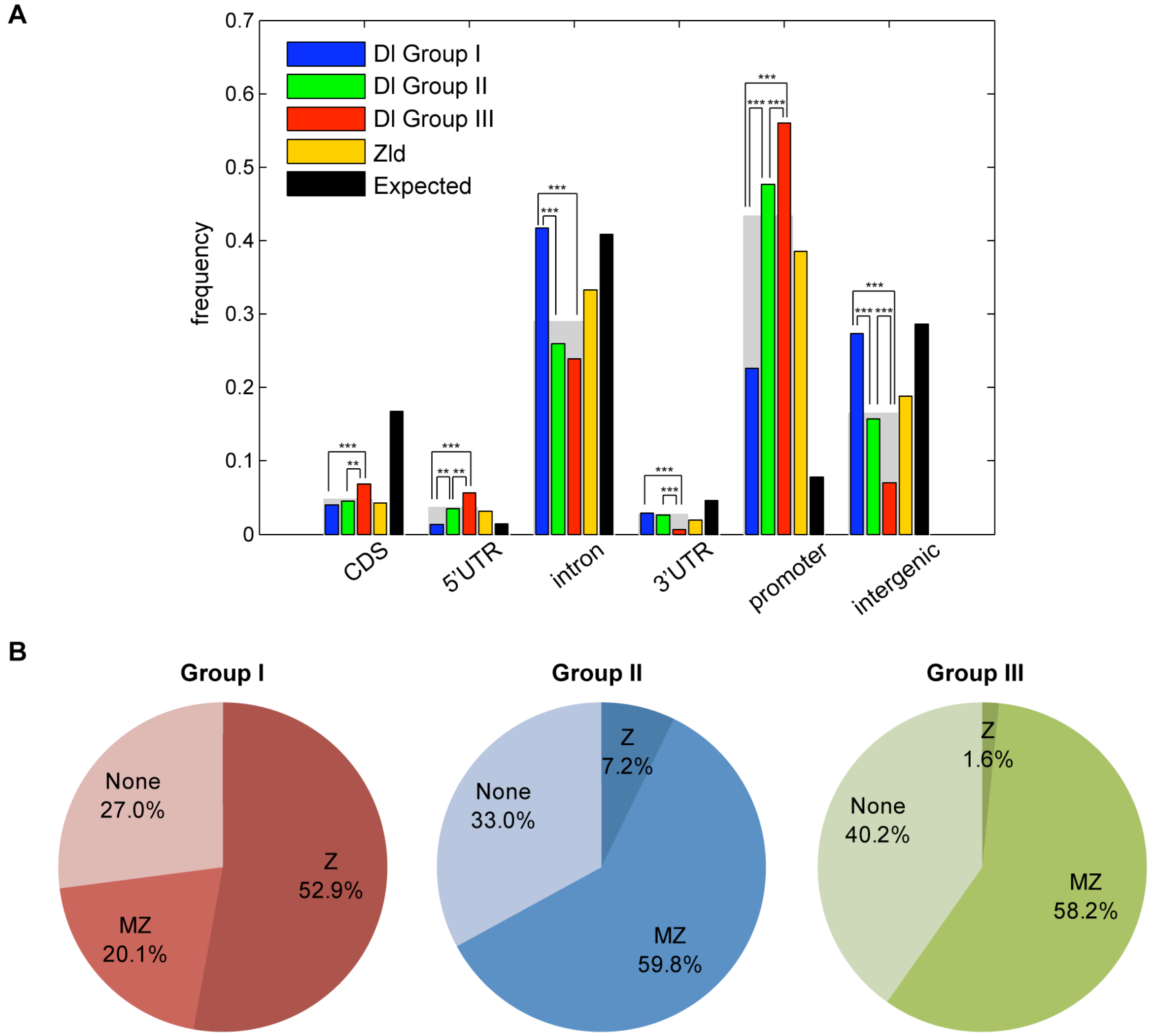

Supplemental Fig. 2. Features of genes assigned to each Dl group. (**A**) Genomic distribution of *wt* Zld peaks, and Dl peaks within each group. The y-axis represents the frequency of peaks belonging to a certain genomic annotation. The expected Dl occurrence for all Dl peaks within each annotation is shown as a gray shadow. The expected percentage of each annotation across the genome is denoted with a black bar. Significantly different annotations between Dl groups are denoted by an asterisk (* p<0.05, ** p<0.001, *** p<0.0001, hypergeometric test). "Promoter" is -500bp to +150bp of a TSS. (**B**) The maternal/zygotic contribution of genes assigned to each Dl group is based on the classification of Chen et al. (Chen et al., 2013), with "Z" indicating actively transcribed zygotic genes (pre-MBT + MBT active), "MZ" indicating genes with both maternal and zygotic contribution (MBT maternal), "None" indicating genes not zygotically expressed (N/A + MBT poised).

Sun_Supplemental Fig3

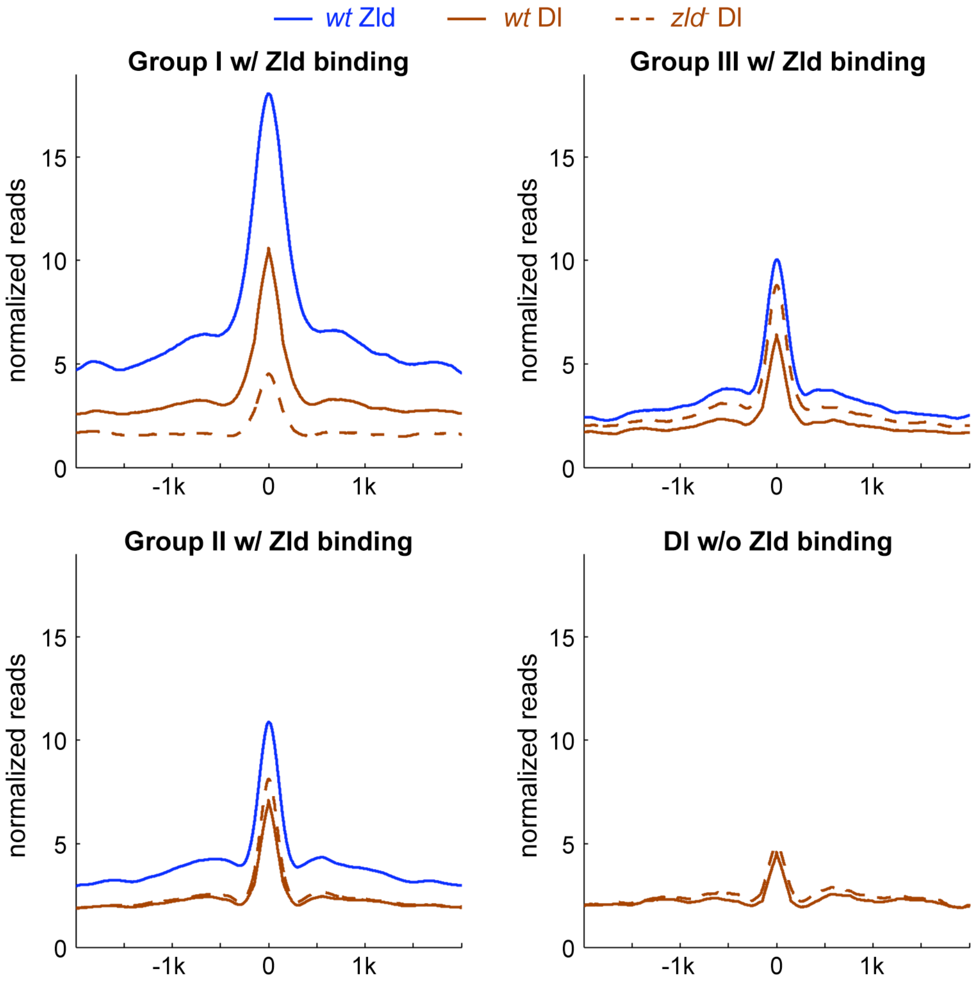

Supplemental Fig. 3. Meta-profiles of Dl peaks that are >1kb away from a TSS are shown for the three Dl-peak groups bound by Zld, as well as Dl peaks that do not co-localize with Zld binding as control. *wt* Zld binding in blue, *wt* Dl binding in solid brown line, *zld*$^{-}$ Dl binding in dashed brown line. The normalized reads were aligned at the Dl summit, and average reads within 2kb distance are shown.

Sun_Supplemental Fig4

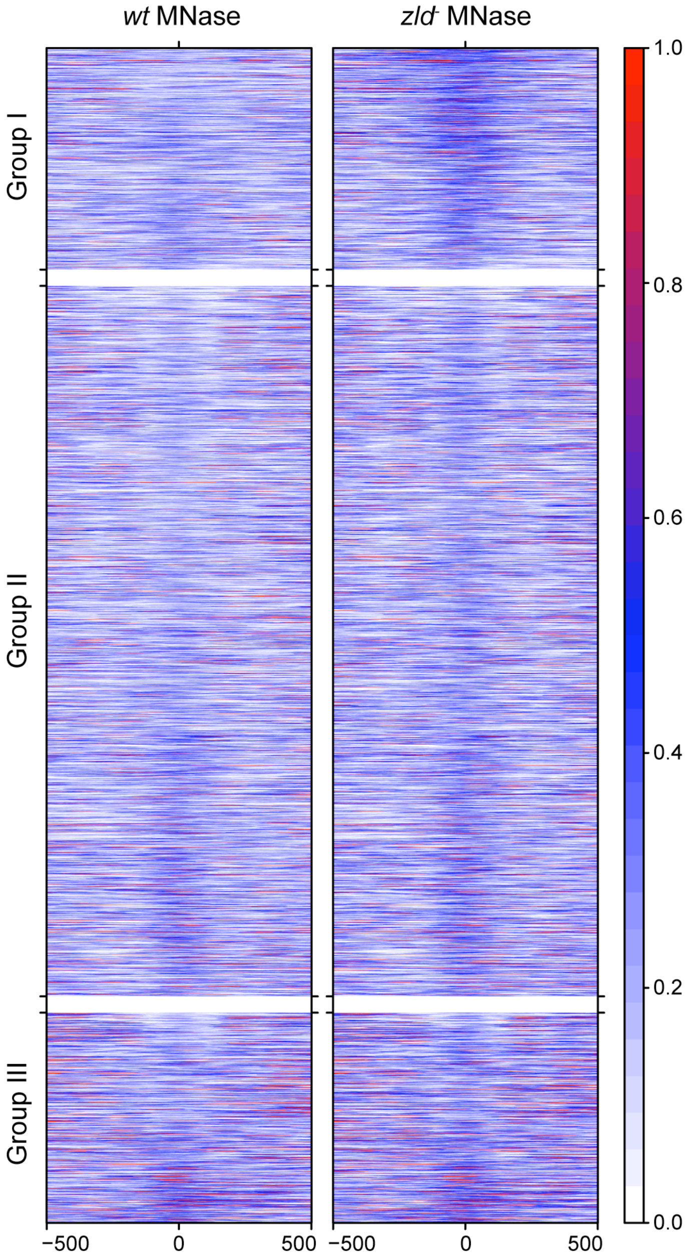

Supplemental Fig. 4. Heatmaps of nucleosome occupancy at 3 Dl groups in *wt* and *zld-* embryos. MNase reads comparing *wt* and *zld-* embryos are shown for the 3 Dl groups, aligned at Dl summit and ranked by *wt* Dl summit reads high to low. The read coverage is in linear scale ranging from minimum (zero reads) to maximum (read value at the 99th percentile among all displayed bases). The x-axis indicates the distance from Dl peak summit (bp). Note within Group I, there is a significant increase in nucleosome occupancy in *zld-* compared to *wt*. For Groups II and III, the overall nucleosome occupancy is comparable between *wt* and *zld-*, indicating that Zld does not have a significant influence on the nucleosome occupancy at these regions.

Sun_Supplemental Fig5

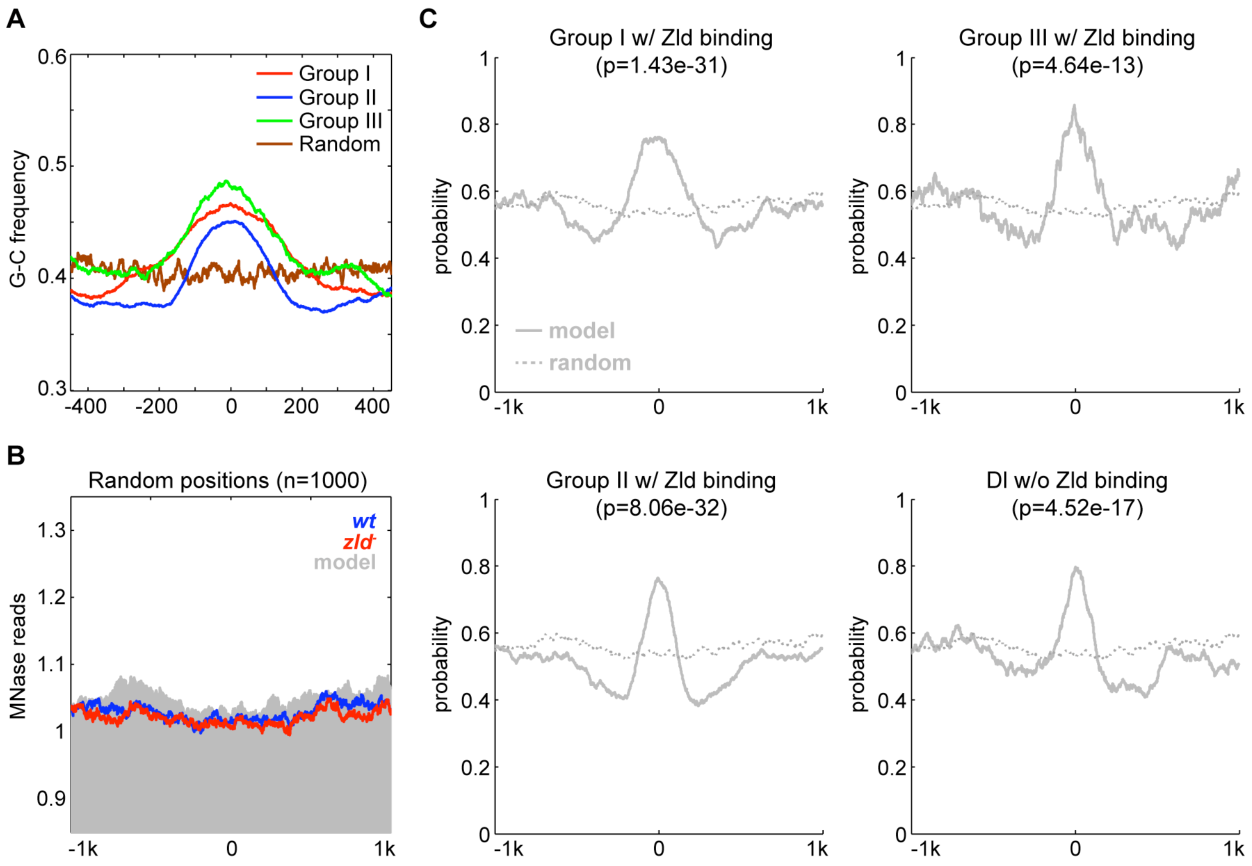

Supplemental Fig. 5. (**A**) G-C frequency is plotted for Dl peaks that are >1kb away from a TSS, centered at Dl summits, and 1000 random regions of 800bp length that are >1kb away from a TSS as control, aligned at the center of the random regions. The random regions were selected so that their G-C content is insignificantly different from that of all non-TSS Dl peaks within ±400bp of Dl summits. Regions around Dl summits in all three groups show relatively higher G-C content than their surrounding regions, while random regions have uniform G-C content. (**B**) Meta-profiles of *wt* (blue) and *zld*- (red) MNase reads, as well as predicted average nucleosome occupancy based on the underlying DNA sequence (grey) using a published prediction model (Xi et al. 2010), are shown for aforementioned 1000 random regions, aligned at the center of the random regions. (**C**) Meta-profiles of predicted nucleosome occupancy based on the underlying DNA sequence are shown for Dl peaks that are >1kb away from a TSS (solid grey line), either bound or not bound by Zld, centered at Dl summits, and aforementioned 1000 random regions (dotted grey line), aligned at the center of the random regions. The predicted nucleosome model for all non-TSS Dl peaks, either bound or not bound by Zld, is significantly different from that at random regions within ±75bp of alignment center, with p-value indicated on top of each group (t-test).

Sun_Supplemental Fig6

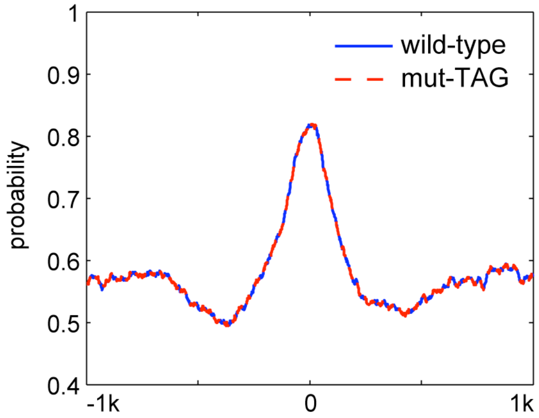

Supplemental Fig. 6. *In silico* Zld motif mutagenesis. All eight Zld motifs (Nien et al. 2011) within Zld bound regions were mutated *in silico* by transition (C ↔ T, G ↔ A). The nucleosome occupancy models based on wild-type DNA sequence (blue) and mutated sequence (red) were predicted with NuPoP (R package; Xi et al. 2010), and the average profiles at top 1000 Zld peaks that are >1kb away from a TSS were plotted, aligned at Zld summits. After mutating Zld motifs, the nucleosome prediction algorithm still predicted high nucleosome occupancy.

Sun_Supplemental Fig7

Experiment	Total reads	Mapped reads	Coverage
wt Dl ChIP Rep1	32,245,179	23,433,272	91.49%
wt Dl input Rep1	25,304,120	18,434,455	91.12%
wt Dl ChIP Rep2	27,668,522	20,263,763	91.61%
wt Dl input Rep 2	26,337,208	19,179,796	91.63%
zld^- Dl ChIP Rep1	33,008,560	22,116,976	91.50%
zld^- Dl input Rep1	25,138,375	17,539,536	91.65%
zld^- Dl ChIP Rep2	29,125,647	20,425,616	89.52%
zld^- Dl input Rep2	27,203,754	19,120,525	92.00%
wt Zld ChIP Rep1	9,647,361	5,589,244	84.50%
wt Zld input Rep1	24,616,671	16,309,411	91.20%
wt Zld ChIP Rep2	113,969,589	85,895,299	91.32%
wt Zld input Rep2	17,145,271	13,081,018	91.53%
gd^7 Zld ChIP Rep1	13422849	9,738,885	88.31%
gd^7 Zld input Rep1	18920890	11,129,487	90.71%
gd^7 Zld ChIP Rep2	137,666,689	93,343,236	88.90%
gd^7 Zld input Rep2	19,094,553	13,959,054	91.37%
wt MNase Rep1	66,488,782	39,998,542	98.51%
wt MNase Rep2	59,379,260	35,062,208	98.47%
zld^- MNase Rep1	74,350,274	44,783,514	98.67%
zld^- MNase Rep2	68,845,544	38,921,312	98.69%

Supplemental Fig. 7. Numbers of total reads, mapped reads and coverage are listed for all ChIP-seq and MNase-seq experiments. The numbers of total reads and mapped reads are calculated by SAMtools (Li et al. 2009). Reads are either extended to the average insert size of the library estimated by Bioanalyzer for ChIP-seq, or extended to the corresponding paired-end tag for MNase-seq, then piled up by customized R-script. The percentage of coverage of each experiment was calculated as total length of regions without zero read divided by total length of the genome.

Sun_Supplemental Fig8

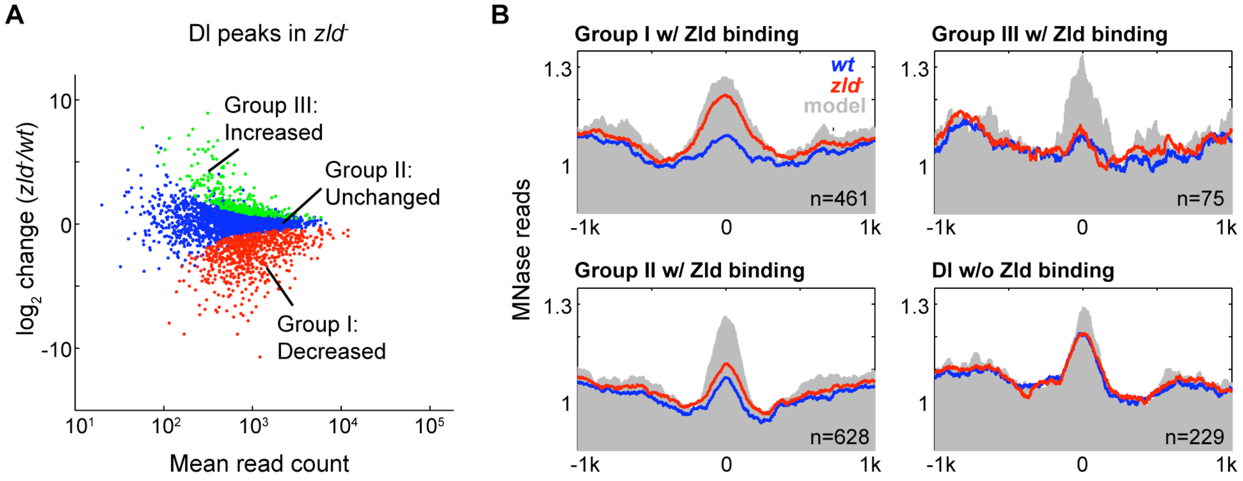

Supplemental Fig. 8. As a normalization control, Dl ChIP and input data were normalized to the mean of total reads, then the differential Dl binding between *wt* and *zld*⁻ was analyzed by DESeq as aforementioned in the main text, and MNase meta-profiles as well as predicted nucleosome model were plotted for each Dl group >1kb away from a TSS. This normalization method generated very similar properties of Dl bound regions and MNase profiles as those generated by our Z-score transformation method used in the main text. (**A**) MA plot of differential Dl binding in *zld*⁻ versus *wt* embryos. The x-axis represents the mean of normalized Dl reads per peak; the y-axis represents the $\log_2$ fold-change of normalized reads per peak between the genotypes. Significantly decreased peaks (Group I, red), not significantly changed peaks (Group II, blue) and significantly increased peaks (Group III, green) were identified by DESeq with FDR<0.1. (**B**) MNase meta-profiles (*wt* in blue, *zld*⁻ in red, predicted nucleosome occupancy model in grey) of Dl peaks that are >1kb away from a TSS are shown for the three Dl-peak groups defined from (A), as well as Dl peaks that do not co-localize with Zld binding as control. The normalized MNase reads and model were aligned at the Dl summit, and average reads (average probability for model) within 1kb distance are shown.

Supplemental Methods

Genomic annotations

Zld and Dl group peaks were each assigned an exclusive genomic annotation based on FlyBase Dmel_Release_5.57 with the following assignment hierarchy: 1) if the peak summit is within a single annotated transcript, it is assigned to the annotations of that transcript; 2) if the peak region has multiple annotations, the peak is assigned to one annotation in the following hierarchical order: promoter (-500bp to +150bp of a TSS), CDS, 5'UTR, 3'UTR and intron; 3) if the peak does not fall into a transcript, it is annotated as in an intergenic region. A peak was considered as "near a TSS" if the peak boundary is within 1kb of a TSS.

Assigning Dl peaks to genes

Dl peaks (from peak summit) were assigned to the nearest TSS based on FlyBase Dmel_Release_5.57. Genes that were assigned to multiple Dl-peak groups were excluded from further analysis.

Maternal/zygotic contribution of genes associated with Dl peaks

The maternal/zygotic contribution of a gene was determined according to Chen et al. (Chen et al. 2013), with "Z" indicating actively transcribed zygotic genes during 1-3h, "MZ" indicating genes with both maternal and zygotic contribution, "None" indicating genes not zygotically expressed (N/A + poised).

Random region control and G-C frequency calculation

1000 random regions of 800bp length were selected across the genome, with the criteria that they are >1kb away from a TSS and that their G-C content is insignificantly different ($p=0.71$, t-test) from that of non-TSS Dl peaks (>1kb away from a TSS) within 400bp of Dl summits. The G-C frequency in Supplemental Fig. 5A within 1kb of the alignment center was calculated with a 75bp sliding window for non-TSS Dl-peak groups and the aforementioned 1000 random regions. In Supplemental Fig. 5C, student's t-test was performed on the predicted nucleosome model centered at Dl summits and 1000 aforementioned random regions within 75bp of alignment center.
